# Long-term monitoring of a brown trout (*Salmo trutta*) population reveals kin-associated migration patterns and contributions by resident trout to the anadromous run

**DOI:** 10.1186/s12862-021-01876-9

**Published:** 2021-07-13

**Authors:** Eloïse Duval, Øystein Skaala, María Quintela, Geir Dahle, Aurélien Delaval, Vidar Wennevik, Kevin A. Glover, Michael M. Hansen

**Affiliations:** 1 Department of Biology, Aarhus University, Ny Munkegade 114, 8000 Aarhus C, Denmark; 2 Department of Aquaculture, Institute of Marine Research, Nordnes, P.O. Box 1870, 5817 Bergen, Norway; 3 Institute of Biology, University of Bergen, Bergen, Norway; 4 Present Address: Theoretical and Experimental Ecology Station, UMR-5321, CNRS, University of Toulouse III Paul Sabatier, 2 route du CNRS, 09200 Moulis, France; 5 Present Address: Faculty of Biosciences and Aquaculture, Nord University, 8049 Bodø, Norway

**Keywords:** Partial migration, *Salmo trutta*, Life-history types, Parentage assignment, Sibship reconstruction, Migration timing, Effective population size

## Abstract

**Background:**

In species showing partial migration, as is the case for many salmonid fishes, it is important to assess how anthropogenic pressure experienced by migrating individuals affects the total population. We focused on brown trout (*Salmo trutta*) from the Guddal River in the Norwegian Hardanger Fjord system, which encompasses both resident and anadromous individuals. Aquaculture has led to increased anthropogenic pressure on brown trout during the marine phase in this region. Fish traps in the Guddal River allow for sampling all ascending anadromous spawners and descending smolts. We analyzed microsatellite DNA markers from all individuals ascending in 2006–2016, along with all emigrating smolts in 2017. We investigated (1) if there was evidence for declines in census numbers and effective population size during that period, (2) if there was association between kinship and migration timing in smolts and anadromous adults, and (3) to what extent resident trout were parents of outmigrating smolts.

**Results:**

Census counts of anadromous spawners showed no evidence for a decline from 2006 to 2016, but were lower than in 2000–2005. Estimates of effective population size also showed no trends of declines during the study period. Sibship reconstruction of the 2017 smolt run showed significant association between kinship and migration timing, and a similar association was indicated in anadromous spawners. Parentage assignment of 2017 smolts with ascending anadromous trout as candidate parents, and assuming that unknown parents represented resident trout, showed that 70% of smolts had at least one resident parent and 24% had two resident parents.

**Conclusions:**

The results bear evidence of a population that after an initial decline has stabilized at a lower number of anadromous spawners. The significant association between kinship and migration timing in smolts suggests that specific episodes of elevated mortality in the sea could disproportionally affect some families and reduce overall effective population size. Finally, the results based on parentage assignment demonstrate a strong buffering effect of resident trout in case of elevated marine mortality affecting anadromous trout, but also highlight that increased mortality of anadromous trout, most of which are females, may lower overall production in the system.

**Supplementary Information:**

The online version contains supplementary material available at 10.1186/s12862-021-01876-9.

## Introduction

Individuals within species can exhibit different life history strategies which are often associated with important phenotypic variation, can differ between sexes and overall have pervasive ecological implications [1]. Hence, individuals representing different life history types may differentially allocate their amount of available energy between maintenance and reproduction functions to maximise their fitness. This polymorphism in life history strategies is maintained within species because their costs and benefits vary according to the environmental contexts [1]. As an example, within many species known to undertake migrations, some individuals migrate while others from the same population remain on the same site across their lifespan, referred to as partial or facultative migration [2, 3].

Among fishes, many salmonid species show anadromous life history forms, which means that juveniles hatch in freshwater and undertake feeding migrations at sea before returning to freshwater for spawning [4]. Their populations often include both sea-migratory and resident individuals that remain in freshwater, therefore showing facultative anadromy [5]. Coexistence between resident and migratory life-history strategies involves a fine balance between their respective costs and benefits [6]. Increased food availability in marine environments may lead to better growth and higher fecundity of anadromous individuals [7, 8]. On the other hand, residency can be advantageous when costs of migration become higher than benefits, due to factors such as predation risk, additional exposure to pathogens and parasites, or energetic costs for the migration process itself [6, 9].

Facultative anadromy is usually considered a quantitative trait, controlled by the action of multiple genes and their interaction with environmental factors [10, 11]. However, recent studies have shown that traits related to migration and life history in some salmonid species can be under control of single genes [12–14], whereas other studies point towards important elements of epigenetic regulation [15]. It is furthermore noteworthy that proportions of migrants and residents within a population may vary across years according to environmental factors or anthropogenically induced disturbances [16–18].

Migratory species, and not least salmonids, may be particularly susceptible to anthropogenic impact due to their dependence on several different habitats and connectivity between them [19]. For instance, fishing pressure in the sea and decreased access to marine environments due to dams represent important issues [20, 21]. Moreover, emerging threats related to climate change altering marine temperature regimes and adverse effects of marine aquaculture, such as accummulation of parasites that subsequently infect wild populations have become increasingly important [22–25]. In addition to general population declines, increased mortality at sea could also disproportionally affect the resident or migratory components of populations showing facultative anadromy. Also, in the case of sex-ratio differences between life-history types [26], increased mortality of one sex could reduce the total effective population size, resulting in a lower ratio between effective and census population size and leading to increased inbreeding and loss of genetic variation [27]. Finally, some studies have suggested that related fish tend to group together during migration [28, 29]. In addition to active association of kin [29], this could also reflect the mere fact that closely related individuals, especially smolts, may be “physiologically timed” to migrate at the same time [30]. If related individuals migrate together and are subjected to specific incidences of e.g., exposure to parasites, this could lead to high variance of survival among families and ultimately increase the variance of their reproductive success, a factor also leading to decreased effective population size [27].

The brown trout (*Salmo trutta*) is a species often showing facultative anadromy within populations [26, 31]. Recent studies have shown that environmental factors such as water temperature and food availability for brown trout juveniles can have contrasting effects on their migration tendency, with food limitation generally favoring anadromy and increasing temperature favoring residency [32–34]. These environmental factors interact with inherited genetic factors to shape life history strategies [33, 35, 36]. Brown trout shows a sex ratio typically skewed towards females among anadromous individuals and towards males among resident individuals, the latter including precocious mature male parr that can successfully fertilize eggs by adopting a sneaking behaviour [5, 26, 37–39]. Fjords in Norway have experienced an increased establishment of Atlantic salmon (*Salmo salar*) farms since the 1990s [40]. The aquaculture industry poses major problems for wild salmon and brown trout during marine migration [41]. Hence, the high concentration of farmed fish attracts and accummulates parasites such as sea lice *Lepeophtheirus salmonis* that subsequently infect wild salmon and trout passing by, often in lethal doses [23, 42–46]. Anadromous brown trout in Norway typically do not migrate beyond a ca. 80 km range from their native river before returning to spawn [47]. Compared to Atlantic salmon, their home range is therefore more restricted to the fjords, where sea lice are concentrated. In some regions, low marine survival of brown trout has indeed been recorded during the last decades and ascribed to increasing exposure to salmon lice [43]. There is also evidence for genetic variation in susceptibility of brown trout populations to salmon lice infestation, further underpinning salmon lice as an important factor in marine mortality [48].

**Fig. 1:**
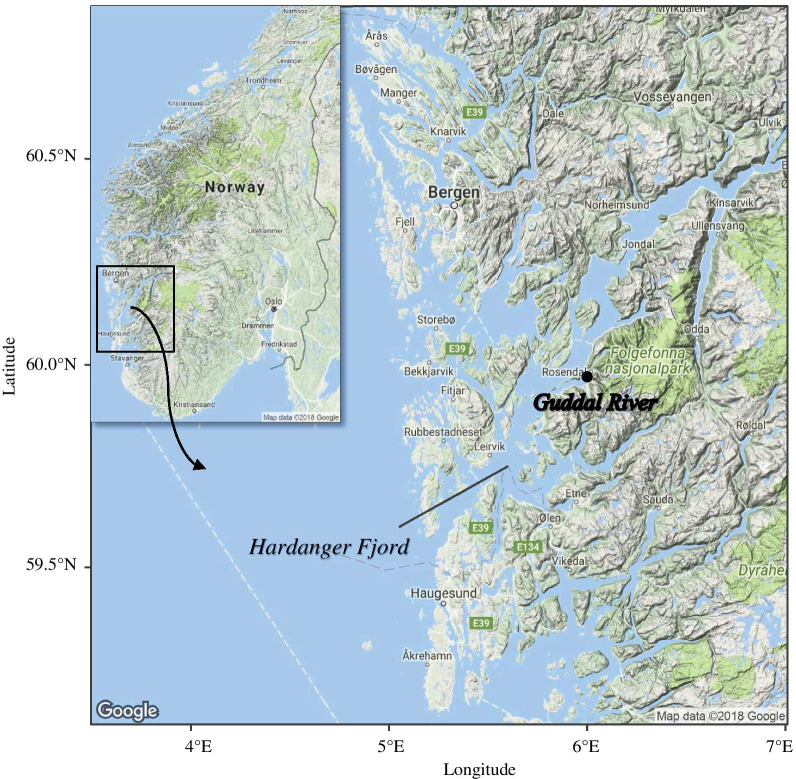
The trap facility is located in the river Guddal, in the central part of the Hardanger Fjord. The black dot indicates the location of the Guddal river mouth. Inset map shows location of the study site in Norway

The present study focuses on the brown trout population of the River Guddal, located in the central region of the Norwegian Hardanger Fjord (Fig. 1). A trap facility encompassing two types of traps was installed in the early 2000’s after a reported decrease in the number of sea trout in the fjord [49]. It allows for a full monitoring of ascending adults and descending smolts, from which phenotypic and genetic data have been secured annually. In turn, this provides a unique long-term data set on the down- and up-stream migratory patterns of brown trout in this river, located in a major farming region where marine survival is known to be low [49].

We analyzed microsatellite DNA variation in all anadromous trout ascending the river from 2006 to 2016 and in all smolts emigrating from the river in 2017. We assessed temporal trends in the number of ascending spawners across years and used genetic data to estimate effective population sizes. We tested the hypothesis that census and effective population sizes had declined over the time span studied. Second, as migratory behaviour in general may involve genetic components [33, 35, 36] and specifically as some heritability has been shown in the timing of migration in other salmonid species [28, 50], we used genetically based parentage assignment and sibship reconstruction to test whether related individuals are more likely to migrate together than non-related individuals. Third, using parentage assignment of smolts from 2017 we assessed the degree to which anadromous and resident trout contributed to the smolt run, thus providing information on the possibility that resident trout can compensate for increased mortality in the marine environment.

## Material and methods

### Study site

The river Guddal (59° 58′ N, 6° 00′ E) is 13.5 km long and located in the central region of the Hardanger Fjord (Fig. 1), the second longest fjord in Norway and showing the highest concentration of Atlantic salmon farms [51]. Survival of wild anadromous trout in the system has been found to be low [43], presumably as a consequence of exposure to sea lice derived from salmon farms, as has recently been determined with genetic studies on lice [46, 52]. The river Guddal is partly fed by melting water running from the Folgefonna glacier, with relatively cold mean summer temperatures ranging from 8.5 to 11.9 °C between 2007 and 2016.

### Sampling

Each year, all smolts descending the river for their first seaward migration are caught in a Wolf trap [53] that covers the whole river transect, located at about 100 m from the tidal zone. The trap is operated annually from March or early April, depending on the water discharge, until the end of the smolt run. In principle, the trap should sample all downstream migrating smolts, but in some years there are shorter periods ranging from a few hours to a couple of days, where water discharge prevents full sampling, so that the trap is estimated to catch 90% of the migrating smolts. Smolts caught in the trap are anaesthetised with benzocaine, measured, weighed, adipose fin-clipped, and tagged with a passive integrated transponder (PIT tags 12 × 2 mm) since 2007 [49, 54] before being released downstream. A specific test showed that among 600 smolts retained in a prolonged recovery, only 4 individuals (0.7%) died (Ø. Skaala, unpublished results). Hence, we do not expect that handling has severely affected the smolts, and any effects should be randomly distributed among individuals and families. In addition, an upstream trap is operated each year during the upstream migration to capture all anadromous trout ascending the river. Each fish is anaesthetised, fin-clipped, checked for PIT tag, measured and weighed before being released upstream to continue its migration. Tissue samples for both smolts and ascending fish were stored in 95% ethanol. For this study, we used data on 711 ascending fish collected between 2006 and 2016 (Table 1), representing all the adults entering the system in this period. Census numbers (but not genetic data) of ascending spawners between 2000 and 2005 were also available [43]. Furthermore, we analysed the entire smolt run of 2017, representing 965 fish (Table 1).

**Table 1:** Summary of brown trout trap sampling and genotyping, both for sea trout ascending the Guddal River in 2006–2016 and for the smolts emigrating to sea in 2017

	Sample	Catches in the trap	Fish with no tissue sample*	Fish identified as salmon	Trout individuals genotyped
Ascending trout	2006	86	0	1	85
	2007	37	3	0	34
	2008	89	7	1	79
	2009	82	1	0	81
	2010	28	1	2	25
	2011	73	3	4	63
	2012	56	0	1	53
	2013	41	2	0	39
	2014	60	1	0	58
	2015	99	0	2	96
	2016	89	2	0	87
Smolt	2017	965	20	94	851

Catches in the trap: total number of ascending fish caught in the trap. * including individuals with too many loci missing after genotyping. Trout individuals genotyped: total number of individuals minus fish with no samples, fish identified as Atlantic salmon and sea trout individuals caught several times within the same year in the ascending trap for adults. Two individuals were caught twice in 2008, two twice in 2011, one individual three times in 2012, one twice in 2014, one twice in 2015 and one twice in 2016

### Genotyping

DNA was isolated and extracted using Qiagen’s DNeasy^®^ Blood & Tissue kit (Qiagen Inc.), following the manufacturer’s recommendations. DNA concentration was measured with a NanoDrop 1000 Spectrophotometer (Thermo Fisher Scientific Inc.). Dilutions were subsequently conducted using a Freedom EVOware^®^ robot (Tecan Inc.), yielding ca. 16.6 ng/µL of DNA per sample.

All samples were genotyped at 21 microsatellite loci (Additional file 1: Appendix 1), divided into three multiplexes for the Polymerase Chain Reaction (PCR). The third multiplex included the sex-specific markers, Exon 2 and Exon 4 [55] from the *sdY* sequence, a male-specific-Y-chromosome gene that is highly conserved in salmonids [56]. Although not always 100% accurate, due to the occasional occurrence of autosomal pseudocopies of *sdY*, at least in brown trout´s closest relative, Atlantic salmon [57], this provides more accurate sexing than phenotypic sex as morphological dimorphism can be difficult to ascertain in the field [58]. PCR was conducted using a Verity 96 well thermal cycler and GeneAmp PCR system 9700 (Applied Biosystems). Conditions for the cycling reactions and primer mixes used for the 3 multiplexes are detailed in Additional file 1: Appendix 2. PCR products were then diluted at 1:15 and separated by capillary electrophoresis using a 3730 DNA Analyzer (Applied Biosystems). Alleles were scored using GeneMapper 5 with GeneScan 500 LIZ size standards (Applied Biosystems). A total of 30 adults and 94 smolts were identified as Atlantic salmon as they showed several allele sizes that do not correspond to alleles known from brown trout, which left a total of 1560 trout sampled (711 adult ascenders and 851 smolt, see Table 1).

Two microsatellite loci (Ssa85 and Ssa197) were represented twice in separate multiplexes in order to provide a control of the allele scoring (Additional file 1: Appendix 1). PCR was repeated for each sample until data were obtained for most loci. In the whole dataset, 3 individuals had missing data at 3 loci, 4 at 2 loci and 56 at 1 locus, out of the 21 loci genotyped. When several PCRs had been conducted for the same individuals, they were compared and the one with the most clear-cut signal was considered right in case of conflict. The locus BG935488 was excluded from the dataset due to difficulties with reliable scoring of alleles. As loci MHC-I and Sasa-TAP2A are closely linked to loci associated with immune responses and have previously been suggested to be under diversifying selection among trout populations from the Hardanger Fjord [59]; they were omitted from analyses of effective population size but were used for sibship reconstruction (see below).

Tissue samples were accidentally missing for 20 of the ascending trout (Table 1). However, the PIT tag number from a missing fish of 2016 had a match with one captured in 2015, allowing to find its genotype based on the previous sampling.

### Individual identification

The “matches” algorithm implemented in GenAlEx 6.5 [60] was used to identify identical genotypes (allowing for one mismatching locus), corresponding to individuals that had ascended the river Guddal several times among or within years. This procedure was implemented as PIT tags can be lost between captures [54], particularly in females during spawning, thus compromising individual identification.

### Genetic diversity and effective population size

Total number of alleles and allelic richness (A_r_) per locus were computed with the R package diveRsity [61], using R 3.6.1 [62]. Observed (H_O_) and unbiased expected heterozygosity (H_E_), as well as inbreeding coefficient (F_IS_) were computed with GenAlEx 6.5. Tests for Hardy–Weinberg equilibrium were performed with Genepop 4.7 [63] using the following Markov chain parameters: 10,000 dememorisation steps, 1000 batches and 10,000 iterations per batch. False discovery rate (FDR) correction was applied to account for multiple testing [64], using the method by Y Benjamini and Y Hochberg [65] implemented in Myriads 1.1 [66].

Effective population sizes (N_e_) for the ascending sea trout and the smolts were estimated with the linkage disequilibrium method implemented in NeEstimator 2.1 [67], using allele frequencies higher than 0.05. As several cohorts are represented among spawning individuals, we assume that the estimates measure N_e_ rather than N_b_ (the effective number of breeders in a single breeding event [68–70]). A smaller number of cohorts were expected to be represented in the sample of smolts and hence estimates could be shifted more towards estimating N_b_.

### Sibship and parentage assignment

COLONY 2.0.5.1 [71] was used to infer full and half sibships both in the 2006–2016 ascending trout and the smolt 2017 datasets. This software infers all possible relationships (siblings and parentage) of all individuals (all offspring and all candidate parents) simultaneously in a full likelihood framework. Analyses of the ascending trout dataset were conducted with no information on parental genotypes, assuming both male and female polygamy as well as possible inbreeding. The full-likelihood method was used at very high precision for the full likelihood calculation and medium run length together with the options sibship scaling, no updated allele frequencies and no sibship priors. In the analysis of the smolt dataset, the trout ascending in or after 2010 were used as candidate parents. We assumed that when fish were last caught before 2010, they were unlikely to be candidate parents, as smolts at these latitudes leave the river between age 2 and 7 years [72]. Similarly, it is unlikely that reproduction in 2016 would result in smolts sampled in 2017, but several trout ascending in 2016 had also ascended in previous years (see Additional file 1: Appendix 2) and could consequently be parents of smolts as a result of previous spawning events. We made use of this information for providing an empirical assessment of the quality of parentage assignment. We thus predicted that anadromous individuals that had ascended the river in 2016 and were identified as parents of smolts from 2017 should also have ascended the river in previous years.

Three COLONY runs with different random number seeds were used to check the reliability of the results. Only the relationships found in the 3 runs were kept in the inferred pedigree. Allelic dropout rate in the input file was estimated with the PopGenReport R package [73]. Genotyping error rate was set to 0.01 for all loci. Each time, full and half-sibships were inferred from the “best configuration” COLONY output files, which is more accurate than the results found by the pairwise analysis in the files full and halfsib dyads [74].

More than 50% of male parr in a population can be precocious [75–77]. As it would be infeasible to sample all resident trout and not least precocious male parr from the system, we inferred the parentage of resident trout indirectly. Hence, when none or only one of the two parents of a smolt was identified among the anadromous candidate parents, it was assumed that missing parents were part of the resident proportion of the population. This was a realistic assumption as all ascending anadromous spawners are assumed to have been caught in the trap and genotyped (only 8 adults caught in the trap were accidentally not sampled for genotyping between 2010 and 2016, Table 1). Moreover, given sex ratio differences between anadromous and resident trout, we further corroborated this assumption by expecting a higher number of supposedly resident males than resident females.

### Sibship and timing of migration events

To test whether related individuals tend to migrate at the same time (both for ascending adults and descending smolts), Mantel tests were conducted between a distance matrix comprising the sibship previously found between pairs of individuals, coded as 1 for unrelated, 0.75 for halfsibs and 0.5 for fullsibs, and a matrix with the distance in days between their date of capture in the traps. The R package ecodist [78] was used for these analyses, and significance was assessed using 10,000 permutations together with 1000 bootstraps. The test was conducted separately for each year in the ascending spawners. Fish that did not have any siblings within the samples of the year or in the smolt sample were removed from the analyses, in order not to bias the results.

## Results

### Numbers of ascending anadromous spawners

The number of anadromous trout ascending the river Guddal showed pronounced variation among years between 2006 and 2016, with a mean of 64.9 ± 22.9 individuals (± SD), ranging from 25 in 2010 to 96 in 2015, but with no general tendency for a decline over this time period (linear regression: F_(1,9)_ = 0.29, R^2^adj = -0.07, *P* = 0.61; see also Table 1). However, compared to the mean number of anadromous trout recorded in the trap in in 2000–2005 in the same river (100 ± 43 (mean ± SD), [43]) there were indications of a decline over a longer time span, although for the entire period of 2000–2016 the result remained non-significant (linear regression: F_(1,15)=_2.90, R^2^adj = 0.11, *P* = 0.11).

After genotyping, we identified 591 individuals among the 711 tissue samples from returning adult sea trout collected between 2006 and 2016. The matching genotypes showed that 82 individuals ascended the river more than once in the study period. Eight individuals were found returning to the river twice within the same year, and one returned three times in the same year (Table 1). Assignment tests using the simulation option in GeneClass2 [79] provided no evidence that the latter individuals were strayers from different populations, except for a single individual whose multilocus genotype was unlikely to occur based on the allele frequencies observed in the Guddal population (data not shown).

Individuals returned to the river on average 1.19 times, ranging from 1 to 6 times (recaptures are illustrated in Additional file 1: Appendix 3). Individuals ascending multiple times returned within a period of 2.7 ± 1.0 (± SD) years on average (Additional file 1: Appendix 3). During the study period, most of the sea trout ascended the river between mid-July and early September, but the spawning run had its earliest start in 2016 on the 14th of May and the latest end in 2010 on the 25th of November (Additional file 1: Appendix 4). The spawning run could be roughly divided into two peaks of migration for most of the years, one in summer and one in early autumn, but the pattern was not clear-cut (Additional file 1: Appendix 4).

A χ^2^ goodness of fit test revealed that the number of females exceeded number of males among anadromous trout across all years, with sex ratios (females:males) ranging from 1.04 in 2013 to 2.13 in 2009 (Table 2, χ^2^ = 28.2, df = 10, *P* = 0.002).

**Table 2:** Summary of the population genetic statistics, both for anadromous trout ascending the river Guddal in 2006–2016 and for smolts sampled in 2017

	Sample	Sample size	Sex-ratio (Nf:Nm)	Number of alleles	A_r_	H_O_	H_E_	HWE deviations	F_IS_	N_e_ (CI_95%_)
Ascending trout	2006	85	1.43	193	8.14	0.75 ± 0.07	0.75 ± 0.07	1	− 0.012 ± 0.024	99 (60–207)
	2007	34	1.83	169	7.98	0.76 ± 0.08	0.76 ± 0.07	0	0.000 ± 0.040	121 (51–∞)
	2008	79	1.55	196	8.15	0.72 ± 0.07	0.74 ± 0.07	0	0.019 ± 0.040	568 (202–∞)
	2009	81	2.12	209	8.28	0.73 ± 0.07	0.74 ± 0.07	0	0.005 ± 0.027	280 (137–2854)
	2010	25	1.78	159	7.64	0.75 ± 0.09	0.76 ± 0.08	0	− 0.007 ± 0.044	3646 (94–∞)
	2011	63	1.25	187	8.16	0.74 ± 0.07	0.75 ± 0.07	0	0.002 ± 0.030	196 (110–639)
	2012	53	1.21	199	8.46	0.71 ± 0.08	0.76 ± 0.08	2	0.049 ± 0.039	275 (96–∞)
	2013	39	1.05	169	7.84	0.72 ± 0.08	0.76 ± 0.07	0	0.032 ± 0.039	108 (55–598)
	2014	58	1.42	191	8.17	0.73 ± 0.08	0.74 ± 0.08	0	− 0.005 ± 0.040	83 (45–252)
	2015	96	1.18	192	8.02	0.74 ± 0.07	0.74 ± 0.07	0	− 0.010 ± 0.025	135 (78–332)
	2016	87	1.42	203	8.23	0.73 ± 0.07	0.75 ± 0.07	0	0.010 ± 0.024	149 (93–313)
Smolt	2017	851	2.15	287	7.32	0.74 ± 0.04	0.75 ± 0.04	17	0.020 ± 0.006	51 (46–56)

N: number of individuals sampled, Nf:Nm: number of females divided by number of males, Ar: allelic richness, H_O_: mean observed heterozygosity, H_E_: mean unbiased expected heterozygosity, F_IS_: mean individual inbreeding coefficient. H_O_, H_E_ and F_IS_ are followed by their 95% confidence interval. HWE deviations: number of deviations from Hardy–Weinberg equilibrium out of the 18 loci tested, after false discovery rate correction. N_e_: effective population size estimated by the linkage disequilibrium method, lowest frequency used 0.05, CI95%: Jack knife confidence interval, ∞: infinite value

### Genetic variation and effective population size estimated from ascending spawners

A total of three deviations from Hardy–Weinberg equilibrium were found in the microsatellite DNA dataset for annually ascending trout after Benjamini and Hochberg [80] False Discovery Rate correction (Table 2). As they concerned three different loci that were not deviating in other annual samples, they were all retained in the analyses. Mean unbiased expected heterozygosity (H_E_) was high and stable across years, ranging from 0.74 to 0.76, and F_IS_ was not significantly different from 0, except in 2012 and 2013 where it nevertheless remained low (Table 2).

Estimates of mean effective population size (N_e_) showed considerable variation, ranging between 83 in 2014 and 3646 in 2010 (Table 2). The highest N_e_ estimate coincided with the lowest sample size (N = 25) and showed a very wide 95% confidence interval (94–∞), indicating that the point estimate is not informative. Considering only the years with sample sizes > 50 (a total of 8 years), the N_e_ point estimates ranged from 83 to 548, with a mean of 223.1. Similar to the number of spawners per year, there was no evidence for a temporal decline of N_e_.

### Genetic variation and effective population size estimated from 2017 smolts

Mean unbiased expected heterozygosity (H_E_) was similar to that observed in anadromous spawners (Table 2). In contrast, however, 17 deviations from Hardy–Weinberg equilibrium were found among the 18 loci analysed (Table 2), likely reflecting the large number of full- and half-sibs (see below) and essentially violating the criterion for Hardy–Weinberg equilibrium of infinite population size, combined with very high statistical power due to the sample size of N = 851. Sex-ratio in the total sample of downstream migrating smolts was more biased towards females than for the anadromous spawners (2.15 against a mean of 1.48 in the spawners, Table 2). The point estimate of effective population size (N_e_) was 51, much lower than estimates based on anadromous spawners (Table 2).

### Relatedness and timing of migration events

Among the 851 smolts migrating to the sea in 2017, the consensus pedigree achieved after three different COLONY runs identified 3198 full and 11,065 halfsib dyads. This represents 317 different fullsib families, with a mean of 2.7 offspring per family (ranging between 1 and 42). The Mantel test between distance matrices composed of distance in Julian date of seaward migration and distance in inferred sibship relation yielded a significant positive correlation for the 815 smolts which had siblings in the run (*r* = 0.027, *P* = 0.0002, Table 3).

**Table 3:** Mantel test results between sibship and distance in week of ascendance for ascending trouts between 2006–2016 and between Julian date of seaward migration for 2017 smolt run

Sample	N	% Fullsib	% Halfsib	% Nonrelated	Mantel’s r	*P*
2006	47	0.65	5.92	93.43	0.112	**0.0040**
2007	12	1.52	10.61	87.88	0.225	**0.0425**
2008	27	0.28	4.84	94.87	0.092	**0.0400**
2009	43	0.00	3.32	96.68	0.023	0.2229
2010	–	–	–	–	–	–
2011	19	0.58	5.85	93.57	-0.007	0.5484
2012	7	4.76	19.05	76.19	0.182	0.1864
2013	17	1.47	5.88	92.65	-0.004	0.4909
2014	27	2.85	4.56	92.59	-0.025	0.6676
2015	56	1.36	4.29	94.35	0.055	**0.0405**
2016	44	1.90	3.07	95.03	-0.010	0.5978
smolt 2017	815	0.96	3.33	95.7	0.027	**0.0002**

Only individuals that were found to have kin within the samples were kept

N: number of individuals that had kins within the sample, %…: percentage of the total matrix that were full, halfsib or non related pair of individuals, Mantel’s r: Pearson correlation between the two matrices, P: p-value for the test. In 2010, no ascenders were found to be related so the Mantel test could not be conducted

For the samples of anadromous spawners, the numbers of individuals with half- or full-sibs in the spawning run of the same year ranged from 0 in 2010 to 56 in 2015 (Table 3). Mantel tests for association of sibship and migration timing conducted for ascending anadromous trout year by year used the week of ascendance in the matrix, as the time window for the ascending migration is larger than for descending migration of smolts. The results were significant for the adults ascending in 4 years (2006: *r* = 0.112, *P* = 0.0040; 2007: *r* = 0.225, *P* = 0.0425; 2008: *r* = 0.092, *P* = 0.0400, and 2015: *r* = 0.055, *P* = 0.0405; Table 3), but not for individuals ascending in the other years.

### Resident trout contribution to anadromous trout run

Parents of the 2017 smolt run were found among the anadromous trout ascending the river Guddal between 2011 and 2016. Among 140 candidate anadromous fathers and 175 candidate anadromous mothers, 33 males and 52 females contributed to the total smolt sample. Among these individuals, four males and 12 females ascended the river in 2016, but as stated previously, the 2017 smolt run would be unlikely to include offspring from the previous year. In accordance with this, all but one of these individuals had in fact been recorded as ascending the river and presumably spawning in previous years, thus lending indirect empirical support to the robustness of parentage assignment. The putative parent recorded in 2016 but not found to ascend the river in previous years was a male. In addition to the possibility of incorrect parentage assignment, it is possible that the male reproduced as mature male parr and smolted and undertook migration to the sea afterwards. It is also possible that this male represents some of the individuals that were accidentally not genotyped (see Table 1).

**Fig. 2:**
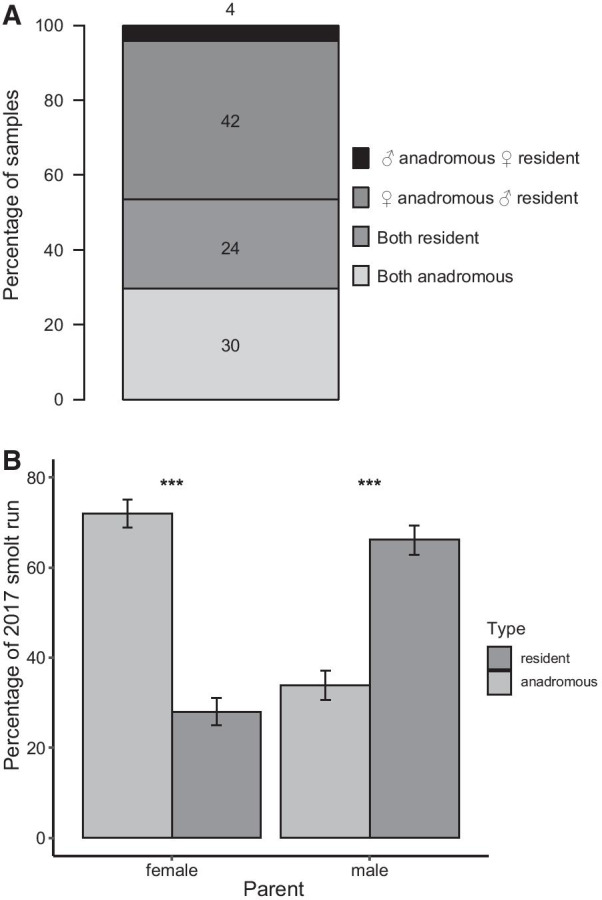
The parental origin of smolts in the 2017 run based on parentage assignment using COLONY 2.0.5.1 [71]. **A** Percentage of smolts according to their parental origin. **B** Contribution of sexes of anadromous and resident trout to the smolt run. Error bars represent the 95% confidence interval of the percentage. Significance of the binomial tests to compare contribution of anadromous and resident individuals of the same sex, ***: *P* < 0.001

Based on the assumption that a parent not represented by anadromous spawners caught in the trap must have been a resident individual, the freshwater resident part of the population was inferred to contribute to 70% of the 2017 smolt run (Fig. 2A). Identified anadromous females contributed to 72% of the smolt sample. The opposite pattern was observed for males, with putatively resident males (including precocious male parr) contributing to 66% of the smolt sample, which was significantly more than anadromous males (binomial tests, N = 851, *P* < 0.001, Fig. 2B). These patterns are in accordance with the skewed sex ratio observed among anadromous spawners (Table 2). A total of 46% of the smolts had one putatively resident parent while the other was a migrant, 24% had two resident parents and 30% had two anadromous parents (Fig. 2A). Among these matings involving two different life history strategy types, 91% took place between a migrant female and a putatively resident male (Fig. 2A).

## Discussion

Our study represents a unique long term monitoring effort of a salmonid population in an environment experiencing significant anthropogenic pressure. In addition to providing insights into demographic trends, the results raised the interesting possibility of concordance between migration timing and kinship in smolts and adult spawners. Finally, and most importantly, parentage assignment of an entire cohort of migrating smolts showed that resident parents contribute substantially to the anadromous run. We discuss these findings and their conservation implications in more detail below.

### Temporal trends of census and effective population size

The number of anadromous trout ascending the Guddal River varied considerably among years, but did not show a general tendency for decline over the study period, although numbers of spawners were lower in 2006–2016 than in 2000–2006. Salmon farming in the Hardangerfjord accellerated during the 1990s, so it is expected that the most severe population declines occurred during that period and have subsequently stabilized, as indicated by our results.

Similar to census sizes of anadromous spawning run, effective population size estimates also showed no general trends towards declines during the study period. This comes with the caveat that estimates of N_e_ based on linkage disequilibrium are sensitive to low sample sizes [81], which for some years/samples resulted in low precision of estimates. It may seem surprising that the N_e_ estimate based on smolts (51) was much lower than any of the estimates based on anadromous spawners. However, as noted previously this estimate is not completely comparable to estimates from adult anadromous spawners that represent different numbers of sea-winters before they return to the river; the smolt run in a given year is expected to represent fewer cohorts compared to adult spawners and would therefore represent something in between N_e_ and N_b_ (the effective number of breeders in a single breeding event [68, 69]).

The N_e_ estimates of a few hundreds are comparable to most other estimates found in anadromous brown trout populations using temporal or LD-based methods [59, 82–84], but higher than most estimates from strictly resident populations [85–88]. Whereas N_e_ in the Guddal population is lower than the 500 or even 1000 assumed to be required for maintaining evolutionary potential [89–92], it should be noted that it is part of a larger system in the Hardanger Fjord where gene flow occurs among populations [59]. In general, anadromous brown trout populations have been found to exhibit a hierarchical genetic structure shaped by both geographical distance between populations and environmental parameters, with low genetic differentiation among local populations resulting from gene flow [93]. Evolutionary potential should thus be considered across several neighbouring populations, where total N_e_ is expected to be higher [94].

### Concordance of migration timing of related individuals

Association of kin along with the possibility of kin selection has been studied intensively in salmonids [29, 95–99]. As sibs during the earliest life stages are situated in the same spawning redds, spatial association of kin would be expected to occur immediately after hatching, whereas subsequent drift and dispersal would lead to decreased kin association over time, unless active kin recognition and association takes place [99]. The significant association betwen migration timing and relatedness as observed in smolts in the present study can hardly be ascribed to reminiscent patterns of association of kin several years back in time in their spawning redds, but could reflect: (1) active aggregation of kin, (2) genetic components in the timing of smolt migration, and/or (3) similarity in size of sibs and thereby propensity for migrating at the same time, simply because sibs hatched and emerged from the same redds at the same time. Whereas the study does not allow for distinguishing between these possibilities, we note that (2) and (3) are the most parsimonious explanations and also indirectly supported by empirical results [47, 100, 101], including data from Atlantic salmon demonstrating clear genetic components in migration timing [96, 102].

Interestingly, our results are at odds with those from a different study on migration timing and kinship in Atlantic salmon, which found no significant association between kinship and schooling and migration timing in smolts [103]. Part of the reason for the discrepancy of results could lie in different experimental set-ups. The study by Fernandes et al. [103] was based on experimental full-sib families stocked into a natural environment at the same point in time, whereas our study encompassed the total smolt run composed of families naturally spawned and hatched over an extended period of time. This would leave more statistical power in our study for detecting association between kinship and migration timing resulting from similar hatching time and size of sibs, without necessarily involving active kin aggregation or genetic components in migration timing.

We also found some support for association between kin and timing of upstream migration among anadromous trout, although significant associations were observed in only 4 out of 11 years. Few studies of possible kin-biased aggregation of adult salmonid fishes have been conducted, undoubtedly due to challenges with sampling. However, one study found kin associations to occur at the adult stage outside the spawning period in brook char (*Salvelinus fontinalis*) inhabiting a large freshwater lake [29]. Nevertheless, kinship analysis of spawners in a tributary to the same lake provided no evidence for association of kin [104]. As noted by the authors, this could be an effect of accummulated mortality over time, leaving few surviving related individuals at the time of spawning. This could also be the case in our present study, where numbers of anadromous spawners per year were overall low.

In total, there was evidence for association between kinship and migration time in smolts, and also evidence, albeit less consistent, for such an association in spawners returning to the river. The association found in smolts raises the possibility that episodes of increased marine mortality, e.g., due to salmon lice exposure or fluctuating aggregations of predators, could potentially increase variance in mortality among families, which could again lead to higher variance in reproductive success among families and lower effective population size. Sibs were found among anadromous spawners in all years except for 2010 (with only 25 ascending anadromous spawners), but whether this reflects a disproportionally high variance in mortality among families compared to undisturbed conditions cannot be assessed. This would require comparable data from the system before major environmental disturbance of the Hardangerfjord system took place.

### To what extent does the resident stock of Guddal brown trout population contribute to the sea run?

In systems like the Hardanger Fjord with increased marine mortality [43–45, 49], it is important to assess to which extent the resident part of the population can compensate for recruitment in the case of a reduced number of anadromous spawners. Moreover, it is important to consider to what extent this will drive changes in anadromy.

We did not genotype candidate parents among the resident trout and made the assumption that non-genotyped parents corresponded to resident individuals. This is an important limitation of the study and for instance precludes distinguishing between parents that are mature male parr and adult resident trout. However, the trap in which ascending spawners were sampled is of a construction that makes it unlikely that individuals can escape further upstream without being registered. In very few instances tissue samples were by mistake not taken, but this is unlikely to account for all the parental genotypes not represented among the ascending anadromous spawners. Moreover, our findings of parentage are in accordance with expectations given the skewed sex-ratio observed among anadromous and resident spawners [5, 26, 37, 39], providing further confidence in our results. Hence, only 4% of all smolts in 2017 had an anadromous father and putatively resident mother, whereas 42% had a putatively resident father and anadromous mother. In total, 70% of the smolts had either one or two inferred resident parents, with the latter category accounting for 24% of all smolts.

In the case of resident males, it is likely that many of them are in fact precocious male parr, as studies of both brown trout and other salmonids have shown that they can be both numerous and have significant reproductive success [6, 38, 39, 105]. On the other hand, the contribution to the 2017 smolt run was higher for anadromous than putatively resident females (72% versus 28%), which could reflect a higher number of anadromous relative to resident females and/or the fact that it is more advantageous as a female to migrate to sea in order to maximize body weight and egg production and thereby reproductive success [31, 106]. In the context of elevated mortality rates at sea, these results demonstrate that resident trout may indeed have some buffering effects towards a decline of anadromous spawners and that a sizeable proportion of smolts in fact have two resident parents. Given the high contribution of anadromous females to the smolt run it is nevertheless also evident that strong declines of the anadromous portion of the population would likely have significant negative demographic consequences for the total population. It should also be stressed that high marine mortality would lead to reduced gene flow among populations, hence reducing overall effective metapopulation size (as in for instance the entire Hardanger Fjord system) and potentially leading to inbreeding and loss of variation in individual populations [94].

Could long-term elevated marine mortality select against anadromy and ultimately remove it from the population? If we assume that anadromy is a quantitative trait with an environmentally-cued threshold [10], then in this case high levels of genetic variation could be preserved even under directional selection acting against it [107]. This way, even if migration costs are increasing, the propensity to migrate within a population may persist. This finds empirical support in studies of brown trout [84] and other salmonid species that have been landlocked for centuries (e.g., by dams) such as brook charr [108], rainbow trout *Oncorhynchus mykiss* [109] and bull trout *Salvelinus confluentus* [110], but where migratory behaviour is retained. Nevertheless, recent studies have demonstrated major quantitative trait loci for important life history and migratory traits in e.g., Chinook *Oncorhynchus tshawytscha* [12] and Atlantic salmon [13]. If such genetically based variation also exists in brown trout, then increased marine mortality could exert strong selection and lead to genetic and phenotypic changes in populations, even if anadromy per se is retained.

## Conclusions

The unique long-term monitoring of ascending anadromous spawners in the Guddal River allowed us to track both census and effective population size over an extended time period coinciding with adverse anthropogenic conditions in the sea. We did not observe general trends of declines during this period, suggesting that the population had stabilized after initial declines prior to the study. We found a significant association between kinship of smolts and their timing of emigration from the river, which raises the possibility that periodically adverse conditions in the sea could disproportionally affect some families and potentially lead to decreased effective population size. A similar association between kinship and migration timing was also indicated in ascending anadromous spawners but was not consistent across years. It is possible that accummulated mortality until this life stage would decrease the number of surviving sibs and thereby weaken signals of an otherwise genuine association. Finally, using parentage assignment of the total smolt run within a year, we estimated that 70% of smolts had at least one resident parent, and in 24% of cases both parents were inferred to be resident. Hence, the resident proportion of the population played a major role in recruitment of anadromous trout and would be expected to provide some buffer against elevated marine mortality. Nevertheless, as the majority of smolts had an anadromous mother, it is also envisaged that elevated marine mortality would have important negative consequences for production of the population as a whole and could also lead to altered selection pressure for important life history traits. In total, the study thus provides important new information about recruitment and dynamics of populations showing partial migration, and how this may interact with anthropogenic environmental disturbance.

## Supplementary Information

**Additional file 1.** Supplementary material on microsatellite loci used (Appendix 1), PCR conditions (Appendix 2), overview of anadromous trout returning to Guddal River several times during the study period (Appendix 3), and proportion of anadromous trout ascending the river in different weeks in specific years (Appendix 4).

## Data Availability

The data that support the findings of this study have been deposited at the Institute of Marine Research electronic archive at: https://hdl.handle.net/11250/2740709. It consists of three text files: a genepop-format file of all the individuals genotyped at 20 microsatellite loci, and two files containing the phenotypic information for adults and smolts, respectively.

## Abbreviations

A_r_: Allelic richness
H_O_: Observed heterozygosity
H_E_: Expected heterozygosity
F_IS_: Fixation index in subpopulations (“inbreeding coefficient”)
FDR: False discovery rate
N_e_: Effective population size
