# Supplementary material for: Long-term monitoring of a brown trout (Salmo trutta) population reveals kin-associated migration patterns and contributions by resident trout to the anadromous run
Source: BMC Ecol Evol. 2021 Jul 13;21:143. doi: 10.1186/s12862-021-01876-9 (PMC8276402; doi:10.1186/s12862-021-01876-9)

**Appendix 1.** Microsatellite loci used in the three different PCR multiplexes.

| Locus | Forward primer | Reverse primer | Multiplex | Number of alleles |
|---|---|---|---|---|
| Ssa85[1] | AGGTGGGTCCTCCAAGCTAC | gtttACCCGCTCCTCACTTAATC | 1, 3 | 13 |
| mOne102a.b[2] | GGGATTATTCTTACTTTGGCTGTT | gtttCCTGGTTGGGAATCACTGC | 1 | 18 |
| Str2QUB[3] | CTGGGGTCCACAGCCTATAA | gtttGAGCTACAACCTGATCCACCA | 1 | 46 |
| Ssa416UoS[4] | TGACCAACAACAAACGCACAT | gtttCCCACCCATTAACACAACTAT | 1 | 4 |
| mOne101[2] | TGCTAAATGACTGAAATGTTGAGA | gtttGAGAATGAATGGCTGAATGGA | 1 | 7 |
| Cocl-lav-4[5] | TGGTGTAATGGCTTTTCCTG | gtttGGGAGCAACATTGGACTCTC | 1 | 11 |
| Oneμ9[6] | CTCTCTTTGGCTCGGGGAATGTT | gtttGCATGTTCTGACAGCCTACAGCT | 1 | 14 |
| CA048828[7] | GAGGGCTTCCCATACAACAA | gtttGTTTAAGCGGTGAGTTGACGAGAG | 1 | 29 |
| BG935488[7] | gtttTGACCCCACCAAGTTTTTCT | AAACACAGTAAGCCCATCTATTG | 2 | unreliable scoring |
| SsaD71[8] | AACGTGAAACATAAATCGATGG | gtTTAAGAATGGGTTGCCTATGAG | 2 | 16 |
| Sasa-TAP2A[9] | gtttGTCCTGATGTTGGCTCCCAGG | GCGGGACACCGTCAGGGCAGT | 2 | 10 |
| MHC-I[9] | AGGAAGGTGCTGAAGAGGAAC | gtttCAATTACCACAAGCCCGCTC | 2 | 19 |
| Ssa410UoS[4] | gtttGGAAAATAATCAATGCTGCTGGTT | CTACAATCTGGACTATCTTCTTCA | 2 | 40 |
| Str3QUB[3] | CTGACCGCTGCACACTAA | gtttGGCTCTAATCGACTGGCAGA | 2 | 12 |
| CA060177[7] | CGCTTCCTGGACAAAAATTA | gtttGAGCACACCCATTCTCA | 2 | 17 |
| Ssa197[1] | GGGTTGAGTAGGGAGGCTTG | gttTGGCAGGGATTTGACATAAC | 2, 3 | 18 |
| SsOsl417[10] | TTGTTCAGTGTATATGTGTCCCAT | GTTTGATCTTCACTGCCACCTTATGACC | 3 | 14 |
| Ssa87NVH[11] | CTGTAAACATCACAGGCG | GTTTCTCCACTAATAGTCTGAAGG | 3 | 16 |
| Ssa24NVH[11] | TGAGTCACACCTGTCACG | GTTTGTGATGATGATTAAAGCCAG | 3 | 6 |
| BS-131[12] | CACATCATGTTACTGCTCC | GTTTCAGCCTAATTCTGAATGAG | 3 | 13 |
| Ssa408[4] | AATGGATTACGGGTACGTTAGACA | GTTTCTCTTGTGCAGGTTCTTCATCTGT | 3 | 28 |

| | | | | |
|---|---|---|---|---|
| Exon2[13] | TGATGGATGGGATCCCCGTCATCTCTCTCCCAAAG | TAGAGCTTAAAACCACTCCACCCTCCATGAGGGA | 3 | - |
| Exon4[13] | AGTTGGAACGCTTCAGCAGAGCAGATGG | AGATTGGTGCACTGAGTGATGAGTCTTGTCC | 3 | - |


1. O'Reilly PT, Hamilton LC, McConnell SK, Wright JM: **Rapid analysis of genetic variation in Atlantic salmon (*Salmo salar*) by PCR multiplexing of dinucleotide and tetranucleotide microsatellites**. *Can J Fish Aquat Sci* 1996, **53**(10):2292-2298.
2. Olsen JB, Wilson SL, Kretschmer EJ, Jones KC, Seeb JE: **Characterization of 14 tetranucleotide microsatellite loci derived from sockeye salmon**. *Mol Ecol* 2000, **9**(12):2185-2187.
3. Keenan K, Bradley C, Magee J, Hynes R, Kennedy R, Crozier W, Poole R, Cross T, McGinnity P, Prodöhl P: **Beaufort trout MicroPlex: a high‐throughput multiplex platform comprising 38 informative microsatellite loci for use in resident and anadromous (sea trout) brown trout *Salmo trutta* genetic studies**. *J Fish Biol* 2013, **82**(6):1789-1804.
4. Cairney M, Taggart JB, Hoyheim B: **Characterization of microsatellite and minisatellite loci in Atlantic salmon (*Salmo salar* L.) and cross-species amplification in other salmonids**. *Mol Ecol* 2000, **9**(12):2175-2178.
5. Rogers SM, Marchand MH, Bernatchez L: **Isolation, characterization and cross-salmonid amplification of 31 microsatellite loci in the lake whitefish ( Coregonus clupeaformis , Mitchill)**. *Mol Ecol Notes* 2004, **4**(1):89-92.
6. Scribner KT, Gust JR, Fields RL: **Isolation and characterization of novel salmon microsatellite loci: Cross-species amplification and population genetic applications**. *Can J Fish Aquat Sci* 1996, **53**(4):833-841.
7. Vasemägi A, Nilsson J, Primmer CR: **Seventy-five EST-linked Atlantic salmon (*Salmo salar* L.) microsatellite markers and their cross-amplification in five salmonid species**. *Mol Ecol Notes* 2005, **5**(2):282-288.
8. King TL, Eackles MS, Letcher BH: **Microsatellite DNA markers for the study of Atlantic salmon (*Salmo salar*) kinship, population structure, and mixed‐fishery analyses**. *Mol Ecol Resour* 2005, **5**(1):130-132.
9. Grimholt U, Drablos F, Jorgensen SM, Hoyheim B, Stet RJM: **The major histocompatibility class I locus in Atlantic salmon (*Salmo salar* L.): polymorphism, linkage analysis and protein modelling**. *Immunogenetics* 2002, **54**(8):570-581.
10. Slettan A, Olsaker I, Lie O: **Atlantic Salmon, *Salmo salar*, microsatellites at the Ssosl25, Ssosl85, Ssosl311, Ssosl417 loci**. *Anim Genet* 1995, **26**(4):281-282.
11. Gharbi K, Gautier A, Danzmann RG, Gharbi S, Sakamoto T, Hoyheim B, Taggart JB, Cairney M, Powell R, Krieg F *et al*: **A linkage map for brown trout (*Salmo trutta*): Chromosome homeologies and comparative genome organization with other salmonid fish**. *Genetics* 2006, **172**(4):2405-2419.
12. Estoup A, Rousset F, Michalakis Y, Cornuet JM, Adriamanga M, Guyomard R: **Comparative analysis of microsatellite and allozyme markers: a case study investigating microgeographic differentiation in brown trout (*Salmo trutta*)**. *Mol Ecol* 1998, **7**(3):339-353.
13. Eisbrenner WD, Botwright N, Cook M, Davidson EA, Dominik S, Elliott NG, Henshall J, Jones SL, Kube PD, Lubieniecki KP: **Evidence for multiple sex-determining loci in Tasmanian Atlantic salmon (*Salmo salar*)**. *Heredity* 2014, **113**(1):86.

**Appendix 2:** Polymerase Chain Reaction conditions. **A**: PCR mixes used for the 3 microsatellite loci multiplexes. Exon 2 and Exon 4 stand for the gender marker primers. Reaction mixes were made in order to get 10µL per well. **B**: PCR programs used for the 3 multiplexes.

**A**

| **Multiplex 1** | Concentration | | **Multiplex 2** | Concentration | | **Multiplex 3** | Concentration | |
|---|---|---|---|---|---|---|---|---|
| DNA | ca 33 | ng | DNA | ca 33 | ng | DNA | ca 33 | ng |
| Buffer | 1 | x | Buffer | 1 | x | Buffer | 1 | x |
| $MgCl_2$ | 2 | mM | $MgCl_2$ | 2 | mM | $MgCl_2$ | 3 | mM |
| dNTP | 0.2 | mM | dNTP | 0.2 | mM | dNTP | 0.2 | mM |
| GoTaq pol | 0.5 | U | GoTaq pol | 0.5 | 1U | GoTaq pol | 0.5 | 1U |
| Ssa85 | 0.2 | mM | BG935488 | 0.2 | mM | Ssa85 | 0.125 | mM |
| mOne102a_b | 0.4 | mM | SsaD71 | 0.5 | mM | SsOsl417 | 0.2 | mM |
| Str2QUB | 0.4 | mM | Sasa-TAP2A | 0.6 | mM | Ssa87NVH | 0.15 | mM |
| Ssa416UoS | 0.4 | mM | MHC-I | 0.3 | mM | Ssa24NVH | 0.3 | mM |
| mOne101 | 0.4 | mM | Ssa410UoS | 0.7 | mM | BS-131 | 0.3 | mM |
| Cocl-Lav-4 | 0.4 | mM | Str3QUB | 0.15 | mM | Ssa408 | 0.3 | mM |
| One_u9 | 0.4 | mM | CA060177 | 0.8 | mM | Ssa197 | 0.125 | mM |
| CA048828 | 0.4 | mM | Ssa197 | 0.2 | mM | Exon2-Vic | 0.05 | mM |
| | | | | | | Exon4-Vic | 0.05 | mM |

**B**

**Multiplexes 1 & 2**

95°C 15min

95°C 45sec
55°C 1min30sec } x5
72°C 1min

95°C 45sec
57°C 1min30sec } x22
72°C 1min

60°C 30min

4°C ∞

**Multiplex 3**

94°C 5min

94°C 45sec
55°C 1min30sec } x3
72°C 1min

94°C 1min
57°C 1min30sec } x25
72°C 1min

72°C 10min

4°C ∞

**Appendix 3:** Sea trout returning to Guddal River several times during the study period. Recaptures indicated for 2017 are based on PIT tags only as no genotypes were available at this time. **A**: Females. **B**: Males.

A

| **Label** | **Years** 2006 | 2007 | 2008 | 2009 | 2010 | 2011 | 2012 | 2013 | 2014 | 2015 | 2016 | 2017 |
|---|---|---|---|---|---|---|---|---|---|---|---|---|
| AC | ■ | ■ | | | | | | | | | | |
| QQ | ■ | ■ | | | | | | | | | | |
| PPP | ■ | | ■ | | | | | | | | | |
| II | ■ | | | | ■ | | | | | | | |
| IIII | | ■ | ■ | | | | | | | | | |
| JJJJ | | ■ | ■ | | | | | | | | | |
| ZZ | | | ■ | ■ | | | | | | | | |
| N | | | ■ | ■ | ■ | | | | | | | |
| SSS | | | ■ | | ■ | ■ | ■ | | | | | |
| FF | | | ■ | | | ■ | | | | | | |
| E | | | | ■ | ■ | | | | | | | |
| TT | | | | ■ | ■ | ■ | | | | | | |
| WWW | | | | ■ | | ■ | ■ | | | | | |
| TTT | | | | | ■ | ■ | ■ | | | | | |
| DD | | | | | | ■ | ■ | | | | | |
| K | | | | | | ■ | ■ | | | | | |
| KKK | | | | | | ■ | ■ | | | | | |
| HH | | | | | | ■ | | | ■ | ■ | ■ | |
| T | | | | | | ■ | ■ | | ■ | | ■ | |
| OO | | | | | | ■ | ■ | ■ | ■ | ■ | ■ | |
| KK | | | | | | | ■ | ■ | | | | |
| FFFF | | | | | | | ■ | ■ | | | | |
| OOO | | | | | | | | ■ | ■ | ■ | | |
| QQQ | | | | | | | | ■ | ■ | | ■ | |
| Z | | | | | | | | | ■ | ■ | | |
| CCCC | | | | | | | | | ■ | ■ | | |
| DDD | | | | | | | | | ■ | ■ | | |
| SS | | | | | | | | | ■ | ■ | | |
| WW | | | | | | | | | ■ | ■ | ■ | |
| V | | | | | | | | | ■ | ■ | ■ | |
| AB | | | | | | | | | ■ | | ■ | |
| CCC | | | | | | | | | ■ | | ■ | |
| X | | | | | | | | | ■ | | ■ | |
| ZZZ | | | | | | | | | | ■ | ■ | |
| AAAA | | | | | | | | | | ■ | ■ | |
| YYY | | | | | | | | | | ■ | ■ | |
| VVV | | | | | | | | | | ■ | ■ | |
| U | | | | | | | | | | ■ | ■ | |
| L | | | | | | | | | | ■ | ■ | |
| EE | | | | | | | | | | ■ | ■ | |
| G | | | | | | | | | | ■ | ■ | |
| H | | | | | | | | | | ■ | ■ | |
| UUU | | | | | | | | | | ■ | ■ | |
| LL | | | | | | | | | | ■ | ■ | |
| BBB | | | | | | | | ■ | | ■ | ■ | ▒ |
| MM | | | | | | | | | ■ | | ■ | ▒ |
| 196 | | | | | | | | | | ■ | | ▒ |
| 388 | | | | | | | | | | | ■ | ▒ |

**B**

| Label | Years | | | | | | | | | | | |
|---|---|---|---|---|---|---|---|---|---|---|---|---|
| | 2006 | 2007 | 2008 | 2009 | 2010 | 2011 | 2012 | 2013 | 2014 | 2015 | 2016 | 2017 |
| AD | ■ | ■ | | | | | | | | | | |
| AE | ■ | | ■ | | | | | | | | | |
| Q | ■ | | ■ | | ■ | | | | | | | |
| Y | ■ | | | ■ | | | | | | | | |
| EEE | | ■ | ■ | | | | | | | | | |
| EEEE | | ■ | ■ | | | | | | | | | |
| NNN | | | ■ | ■ | | | | | | | | |
| C | | | | ■ | ■ | ■ | ■ | | | | | |
| I | | | | | | ■ | ■ | | | | | |
| LLL | | | | | | ■ | ■ | | | | | |
| NN | | | | | | ■ | ■ | | | | | |
| BBBB | | | | | | | | ■ | ■ | | | |
| F | | | | | | | | ■ | ■ | | | |
| HHH | | | | | | | | ■ | ■ | ■ | | |
| GGGG | | | | | | | | ■ | | ■ | | |
| XXX | | | | | | | | ■ | ■ | ■ | ■ | |
| KKKK | | | | | | | | ■ | | | ■ | |
| W | | | | | | | | | ■ | ■ | | |
| S | | | | | | | | | ■ | ■ | | |
| UU | | | | | | | | | ■ | ■ | | |
| BB | | | | | | | | | ■ | ■ | ■ | |
| CC | | | | | | | | | ■ | ■ | ■ | |
| AAA | | | | | | | | | ■ | | ■ | |
| JJJ | | | | | | | | | ■ | | ■ | |
| AA | | | | | | | | | | ■ | ■ | |
| D | | | | | | | | | | ■ | ■ | |
| GGG | | | | | | | | | | ■ | ■ | |
| III | | | | | | | | | | ■ | ■ | |
| J | | | | | | | | | | ■ | ■ | |
| M | | | | | | | | | | ■ | ■ | |
| PP | | | | | | | | | | ■ | ■ | |
| R | | | | | | | | | | ■ | ■ | |
| 319 | | | | | | | | | | ■ | | ░ |
| VV | | | | | | | | | | ■ | ■ | ░ |
| GG | | | | | | | | | | ■ | ■ | ░ |
| HHHH | | | | | | | | | | ■ | ■ | ░ |
| MMM | | | | | | | | | | ■ | ■ | ░ |

**Appendix 4**: Proportion of anadromous trout ascending Guddal River according to the week number between 2006 and 2017. Individuals from 2017 were not genotyped, so the data might contain some Atlantic salmons as well as individuals that ascended multiple times. Week 20 is mid-May, week 45 beginning of November.

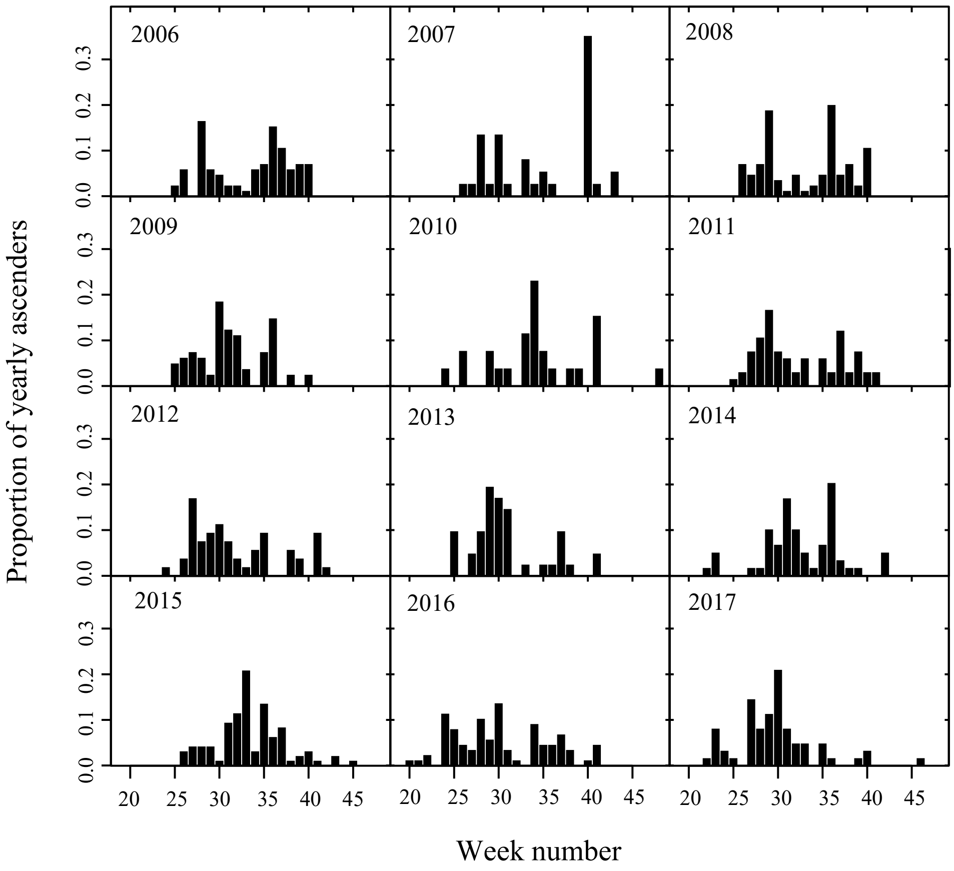

Supplement: Supplementary file 1 — Additional file 1. Supplementary material on microsatellite loci used (Appendix 1), PCR conditions (Appendix 2), overview of anadromous trout returning to Guddal River several times during the study period (Appendix 3), and proportion of anadromous trout ascending the river in different weeks in specific years (Appendix 4). [file 12862_2021_1876_MOESM1_ESM.pdf]
